# Targeting DNA repair for cancer treatment: Lessons from PARP inhibitor trials

**DOI:** 10.32604/or.2023.028310

**Published:** 2023-06-27

**Authors:** DHANYA K. NAMBIAR, DEEPALI MISHRA, RANA P. SINGH

**Affiliations:** 1 Department of Radiation Oncology, Stanford University School of Medicine, Stanford, CA, 94305, USA; 2 Cancer Biology Laboratory, School of Life Sciences, Jawaharlal Nehru University, New Delhi, 110067, India; 3 Special Centre for Systems Medicine, Jawaharlal Nehru University, New Delhi, 10067, India

**Keywords:** PARP inhibitors, Synthetic lethality, DNA repair, BRCA mutations

## Abstract

Ionizing radiation is frequently used to treat solid tumors, as it causes DNA damage and kill cancer cells. However, damaged DNA is repaired involving poly-(ADP-ribose) polymerase-1 (PARP-1) causing resistance to radiation therapy. Thus, PARP-1 represents an important target in multiple cancer types, including prostate cancer. PARP is a nuclear enzyme essential for single-strand DNA breaks repair. Inhibiting PARP-1 is lethal in a wide range of cancer cells that lack the homologous recombination repair (HR) pathway. This article provides a concise and simplified overview of the development of PARP inhibitors in the laboratory and their clinical applications. We focused on the use of PARP inhibitors in various cancers, including prostate cancer. We also discussed some of the underlying principles and challenges that may affect the clinical efficacy of PARP inhibitors.

## Introduction

DNA repair pathways in normal cells ensure error-free replication and maintain genomic integrity. However, it is known that DNA repair genes are frequently mutated in cancer which contributes significantly to cancer development and progression [1]. DNA repair dysfunction is an ideal ally for the cancer cells to acquire an aggressive phenotype and therapeutic resistance [2,3]. Our cells utilize multiple different mechanisms of DNA repair based on the kind of lesion-induced. These include direct repair, mismatch repair (MMR), base excision repair (BER), nucleotide excision repair (NER), and double-strand break (DSB) recombinational repair, which encompasses both non-homologous end-joining (NHEJ) and homologous recombinational repair (HR). This remarkable redundancy in the DNA repair pathway is in place to ensure precision in the process of DNA replication. This also guarantees that the cells have a second chance to survive, even if one of these pathways fails.

During cancer development, the cells continue to acquire different mutations, not only due to defects in DNA repair/genomic instability but also due to poor redox balance in these cells. The poorly functioning redox regulation causes these cells to have higher oxidative stress. The sustained oxidative stress leads to significant oxidative DNA damage, which further contributes to genomic instability and higher mutational burden [4]. In this context, the same backup repair pathways assist them to remain viable and repair the damage caused by chemotherapeutic or other genotoxic agents used for treatment. Given that the cancers with defective DNA repair genes rely on alternative pathways, it was correctly hypothesized that further blocking of the other DNA repair pathways would be lethal for these cells. The concept of “synthetic lethality” [5], which describes a situation “where a defect in one gene is compatible with cell viability but results in cell death when combined with a defect in another gene”, has thus formed the basis of newer targeted therapies especially focusing the DNA repair pathways in cancers [6].

## Exploiting Synthetic Lethality Approach for Cancer Treatment

Though synthetic lethality was established decades earlier, the potential of this approach for drug targeting in cancer was only harnessed in recent years, due to a lack of robust and systematic tools for identifying the synthetic lethal genetic combinations. Recently, genome-wide drug-sensitization screening using short hairpin RNA (shRNA) and small interfering RNA (siRNA)–as well as small-molecule inhibitors have led to the identification of many novel drug candidates (reviewed in McLoran et al.) [7]. The synthetic lethality approach is specifically useful in cancer, as it facilitates the development of cancer-specific cytotoxic agents, which would not affect the “non-mutated” normal cells with a robust repair system. One example of this approach, which has been very successful in the clinic, is the use of PARP (Poly-ADP-ribose polymerase) inhibitors for BRCA mutant cancers.

## The Promise of PARP Inhibitors

**Figure 1:**
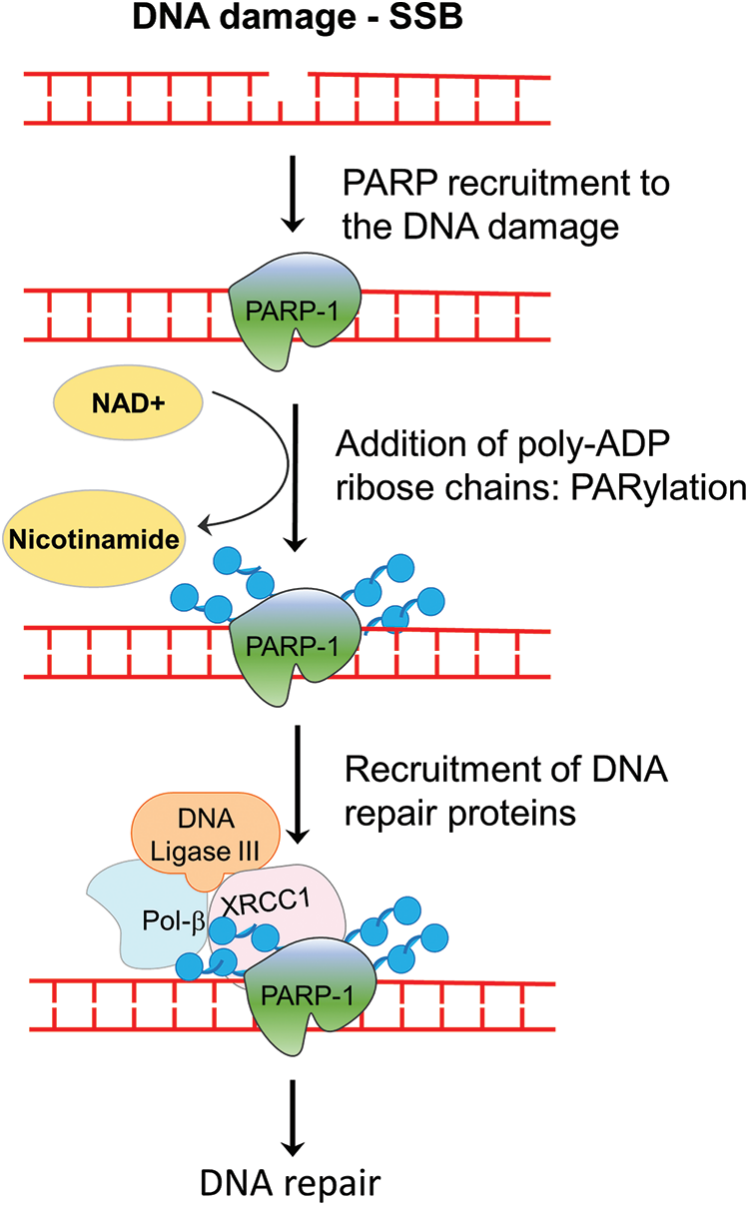
Role of PARP enzyme in DNA repair. In the event of a single-stranded break in cells, PARP enzymes are recruited to the strand break, where it catalyzes the addition of PAR chains on itself or on the other target proteins. PARylation-mediated changes in the chromatin assembly help in the recruitment of repair enzymes, which ultimately repair the break. SSB, single-strand break; NAD^+^, nicotinamide adenine dinucleotide; Pol beta, DNA polymerase beta; PAR, poly-ADP ribose.

**Figure 2:**
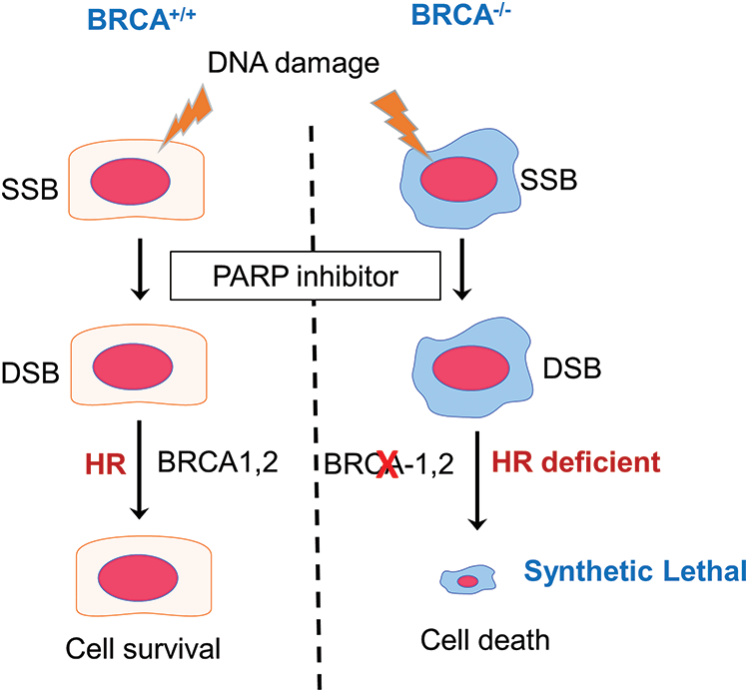
The concept of synthetic lethality in the context of BRCA mutation carriers treated with PARP inhibitors. In the event of DNA damage caused by oxidative base damage, ionizing radiation, and other chemical agents, single-strand breaks (SSBs) are generated. PARP inhibitors block the repair of SSBs, which when left unrepaired are converted to double-strand breaks (DSBs) following replication. In cells with functional *BRCA* genes, these DSB lesions are repaired by homologous recombination (HR) and the cells remain viable. However, in cells that are HR deficient, such as *BRCA* mutant tumor cells, the DSBs cannot be repaired, leading to cell death. PARP inhibition, therefore, is synthetic lethal in HR-deficient cells.

The PARP family of proteins comprised of 17 members which were identified on the basis of their homology in the catalytic domain. PARP1, a nuclear enzyme, is the most prominent member of the family as it accounts for 85% of total PARP activity [8]. PARP enzyme catalyzes the transfer of the first ADP-ribose from nicotinamide adenine dinucleotide (NAD^+^) to the amino acid residues on target proteins and generates a poly-ADP-ribose unit chain (PAR). This process of “PARylation” on proteins as well as on the PARP enzyme itself (self-PARylation) creates a negative charge which reworks the protein structure and function, helping with the binding of multiple proteins (Fig. 1). The PARylation at DNA breaks helps in the recruitment of DNA repair proteins like DNA ligase 3, XRCC1, DNA polymerase β as well as the MRE11-Rad50-NBS1 (MRN) complex [9,10]. Therefore, the PARP enzyme function is crucial not only for BER but also in the HR and NHEJ mechanisms. Since, the PARP function is specifically crucial for single-strand break (SSB) repair, inhibiting PARP will lead to persistent SSBs that, when encountered by the replication fork, are converted into double-stranded breaks (DSBs). The repair of DSB would require a functional HR repair pathway. Therefore, cancers with HR repair deficiencies would be highly sensitive to PARP inhibition, as the lesions will remain unrepaired and eventually cause cell death (Fig. 2). This hypothesis was verified by mouse models, where deletion of PARP-1 increased sensitivity to DNA-damaging agents that induce DNA SSBs, without being embryonic lethal [11,12]. Multiple studies using PARP inhibitors in various tumor models found that PARP inhibitors could sensitize tumor cells to cytotoxic therapies such as temozolomide, topoisomerase I inhibitors, platinum-based chemotherapeutics, and radiation treatment [13–15].

**Figure 3:**
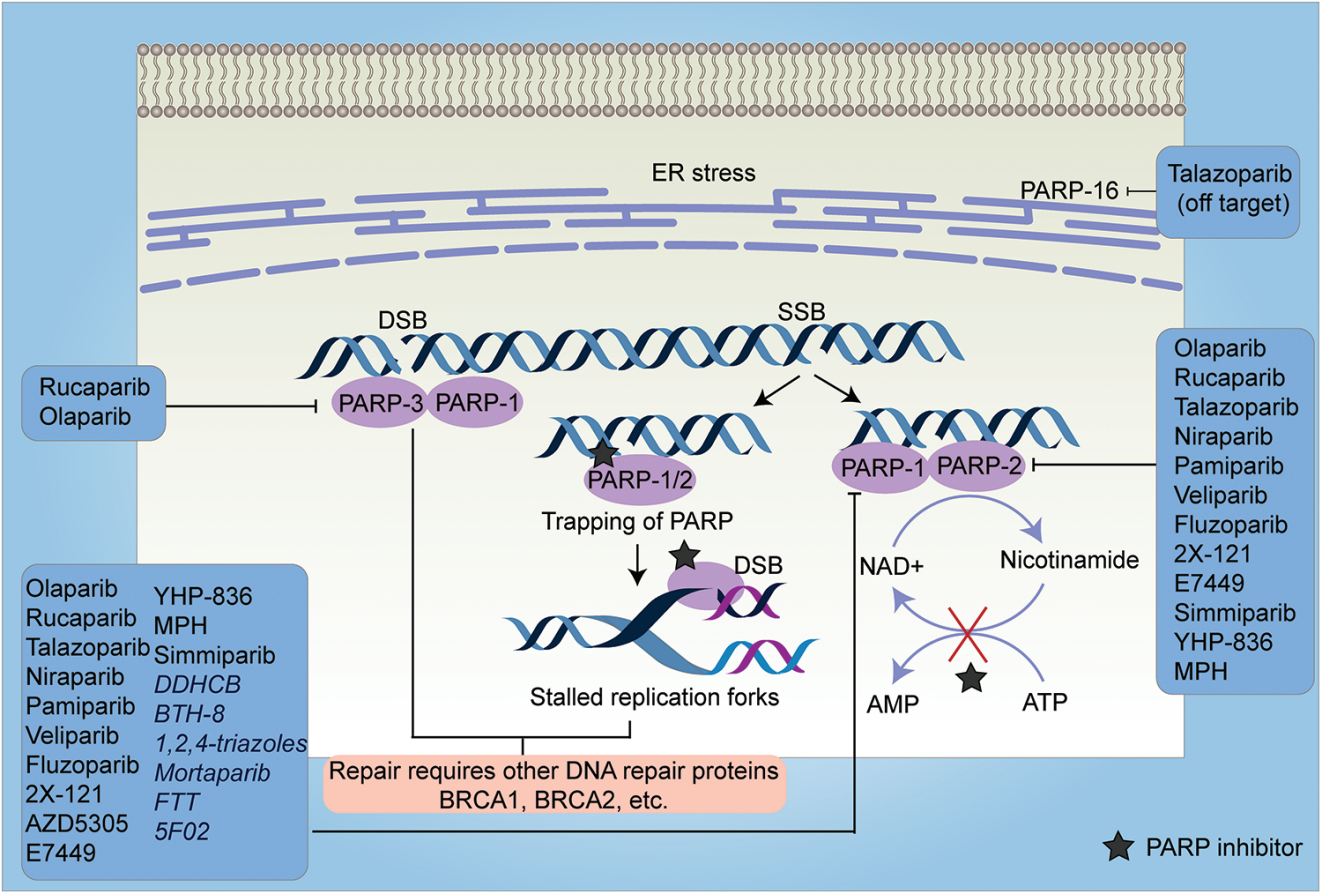
A schematic representation of the mechanism of action of PARP inhibitors and their targets. PARP inhibitors work either by inhibiting the PARylation reaction (except 5F02 which is a non-NAD-like PARP inhibitor) or by trapping the PARP enzyme at the DNA lesions. Trapping of PARP at single-strand breaks (SSBs) causes stalled replication forks which in turn leads to the production of double-strand breaks (DSBs). In HR-efficient cells, these lesions are repaired, however, in HR-deficient cells, it leads to cell death. Most of the PARP inhibitors target either PARP-1, 2, and 3 (inhibition of PARP-16 is off-target effect of Talazoparib) or PARP-1 and PARP-2 or only PARP-1. The inhibitors which exclusively target PARP-1 are depicted in italics. SSB-Single Strand Breaks, DSB-Double Strand Breaks, ER-Endoplasmic Reticulum, FTT-[18F]FluorThanatrace, MPH-(Mefuparib Hydrochloride).

With a better understanding of SSB repair and homologous recombination (HR) repair-mediated DSB repair, two seminal studies applied the approach of synthetic lethality with promising results, as they demonstrated the potential of poly-(ADP-ribose) polymerase (PARP1) inhibition in treating BRCA-mutant tumors [16,17]. BRCA1 and BRCA2 play a crucial role in the repair of double-stranded breaks (DSBs) by homologous recombination (HR) [18]. Heterozygous germline mutation in the BRCA1 gene confers a 60% lifetime risk of breast or ovarian cancer, whereas BRCA2 mutations are associated with a risk of breast or ovarian cancer of 55% and 15%, respectively [19]. Reasonably, tumors with malfunctioning BRCA genes are deficient in the HR repair pathway. Based on these preclinical studies which showed that cells with dysfunctional BRCA1 or BRCA2 are dramatically more sensitive to PARP inhibitors, multiple PARP inhibitors were tested in *in silico*, *in vitro*, and *in vivo* studies and in clinical trials which are summarized in Tables 1 and 2, respectively and in Fig. 3.

**Table 1:** PARP inhibitors in *in silico*, *in vitro*, and *in vivo* studies for prostate cancer, breast cancer, and ovarian cancer

S. No.	PARP inhibitor	Cancer	Study type	Cell line	Target	References
1	5F02	Prostate cancer	*In vitro* and *in vivo*	PC-3 xenograft	Non-NAD-like PARP-1 inhibitor	[20]
2	Simmiparib	Breast cancer	*In vitro* and *in vivo*	Xenografts, CDX and PDX	PARP-1, PARP-2	[21]
3	DDHCB		*In vitro* and *in vivo*	HCC-1937 cell line xenografts	PARP-1	[22]
4	BTH-8		*In vivo* and *in vitro*, using BRCA-deficient cancer cells	HCC-1937 cell line xenograft	PARP-1	[23]
5	YHP-836		*In vitro* and *in vivo*	MDA-MD-436 cell line xenograft	PARP-1, PARP-2	[24]
6	ZC-22		*In vitro* and *in vivo*	MDA-MD-231 cell line xenograft	PARP and CDK4/6	[25]
7	Mefuparib hydrochloride (MPH)		*In vitro* and *in vivo*	MDA-MB-436 cell line xenograft	PARP-1, PARP-2	[26]
8	1,2,4-triazoles		*In silico* and *in vitro*	MCF-7 cell line	PARP-1	[27]
9	Mortaparib	Ovarian cancer	*In vitro* and *in vivo*	SKOV3 ovarian cancer cells xenograft	PARP-1 and mortalin	[28]
10	ZC-22		*In vitro* and *in vivo*	OVCAR5 Ovarian cancer cells xenograft	PARP and CDK4/6	[25,29]

**Table 2:** PARP inhibitors as monotherapy in various phases of clinical trials, for metastatic castration-resistant prostate cancer (mCRPC), breast cancer, and ovarian cancer

S. No.	PARP inhibitor	Cancer	Main trial	Status	Cohort	Target	References
1	Niraparib	Prostate Cancer	GALAHAD NCT02854436	Under phase 2 trial Trial start: 31 Aug. 2016 Estimated completion: 31 Oct. 2022 Status: active	mCRPC with alternation in DNA repair	PARP-1, PARP-2	[30,31]
2	Talazoparib		TALAPRO-1 NCT03148795	Under phase 2 trial Trial start: 4 Jul. 2017 Estimated completion: 31 Oct. 2024	mCRPC with alterations in DDR-HRR who have received both AR-directed therapy and taxane-based chemotherapy	PARP-1, PARP-2, PARP-16	[32,33]
3	Pamiparib		NCT05327621	Under phase 2 trial Trial start: 01 May 2022 Estimated completion: 20 Mar. 2025	mCRCP with homologous recombination deficiency or BRCA 1 or 2 somatic/germline mutation.	PARP-1 PARP-2	[34]
4	Veliparib (With or without Abiraterone Acetate and Prednisone)		NCT01576172	Phase 2 trial completed on 23 Apr. 2020	mCRPC	PARP-1, PARP-2	[35]
5	Fluzoparib (Alone or with Apatinib)	Breast Cancer	FZPL-III-303 NCT04296370	Under phase 3 trial Trial start: 13 Jul. 2020 Estimated completion: 30 Jun. 2025	BRCA mutated HER-2 negative metastatic breast cancer	PARP-1, PARP-2	[36]
6	Niraparib		BRAVO NCT01905592	Phase 3 trial completed 26 Oct. 2021	BRCA mutated HER-2 negative metastatic breast cancer	PARP-1, PARP-2	[37,38]
			NCT05232006	Under phase 2 trial Trial start: May 2022 Estimated completion: May 2030	Advanced metastatic breast cancer in germline PALB2 mutations carriers		
7	2X-121		NCT03562832	Under phase 2 trial Trial start: 20 Jun. 2018 Estimated completion: Oct. 2022 Status: active	Metastatic breast cancer	PARP-1, PARP-2 and Tankyrase 1/2	[39]
8	Rucaparib		NCT02505048	Phase 2 trial completed on Dec. 2019	Metastatic breast cancer with BRCAness genomic signature	PARP-1, PARP-2, PARP-3	[40]
9	NMS-03305293		NCT04182516	Under phase 1 trial Trial start: 25 Nov. 2019 Estimated completion: 30 Dec. 2023	Patients with advanced solid tumors (including breast cancer)	PARP	[41]
10	AZD5305 (Alone or in combination with anti-cancer agents)		NCT04644068	Under phase 1/2 trial Trial start: 12 Nov. 2020 Estimated completion: 29 Jul. 2025	Patients with advanced solid malignancy (including breast cancer)	PARP-1	[42,43]
11	RP12146		NCT05002868	Under phase 1 trial Trial start: 05 Oct. 2021 Estimated completion: Aug. 2023	Patients with locally advanced or metastatic solid tumors (including locally advanced/ metastatic breast cancer)	PARP	[44]
12	AZD9574 (alone or in combination with anti-cancer agents)		NCT05417594	Under phase 1/2 trial Trial start: 24 Jun. 2022 Estimated completion: 30 Jun. 2025	Advanced cancer that has recurred/progressed (including breast cancer)	PARP	[45]
13	E7449 (Alone or in combination with Temozolomide (TMZ) or with Carboplatin and Paclitaxel		NCT01618136	Phase 1/2 trial completed in Jul. 2015	Patients with advanced solid tumors (including triple-negative breast cancer)	PARP-1, PARP-2 and tankyrase 1/2	[46,47]
14	AMXI-5001		ATLAS-101 NCT04503265	Under phase 1/2 trial Trial start: 12 Aug. 2020 Estimated completion: Jan. 2023	Advanced malignant neoplasm (including breast cancer) who have failed other therapies	PARP and microtubule polymerization inhibitor	[48]
15	Pamiparib		NCT03333915	Under phase 2 trial Trial start: 21 Dec. 2016 Estimated completion: Nov. 2021 Status: active	Chinese patients with triple negative breast cancer	PARP-1, PARP-2	[49]
16	Simmiparib		NCT02993913	Under phase 1 trial for malignant solid tumors Trial start: Dec. 2016 Estimated primary completion: Dec. 2018 Status: unknown	Malignant tumors	PARP-1, PARP-2	[50,51]
17	Veliparib	Ovarian Cancer	VELIA NCT02470585	Under phase 3 trial Trial start: 29 Jun. 2015 Estimated completion: 08 Dec. 2026	With Carboplatin and Paclitaxel and as continuation maintenance therapy in advanced ovarian cancer	PARP-1, PARP-2	[52]
18	IMP4297		NCT04169997	Under phase 3 trial Trial start: 24 Dec. 2019 Estimated completion: 30 Dec. 2022	Advanced ovarian cancer	PARP	[53]
19	Talazoparib		NCT04598321	Under phase 1 trial Trial start: 29 Mar. 2021 Estimated completion: Jan. 2027	BRCA mutated ovarian cancer	PARP-1, PARP-2, PARP-16	[54]
20	E7449 (Alone or in Combination with Temozolomide (TMZ) or with Carboplatin and Paclitaxel		NCT01618136	Phase 1/2 trial completed in Jul. 2015	Patients with advanced solid tumors (including ovarian cancer)	PARP-1, PARP-2 and tankyrase 1/2	[46,47]
21	Pamiparib		NCT05489926	Under phase 2 trial Trial start: 16 Aug. 2022 Estimated completion: Dec. 2023	Epithelial Ovarian Cancer EOC with prior exposure to a PARP inhibitor	PARP-1, PARP-2	[55]
22	Fluzoparib		NCT03509636	Phase 1 trial completed on 23 Jul. 2020	BRCA mutated ovarian cancer	PARP-1, PARP-2	[56,57]
23	AZD5305		NCT04644068	Under phase 1/2 trial Trial start: 12 Nov. 2020 Estimated completion: 29 Jul. 2025	Patients with advanced solid malignancy (including ovarian cancer)	PARP-1	[42,43]
24	RP12146		NCT05002868	Under phase 1 trial Trial start: 05 Oct. 2021 Estimated completion: Aug. 2023	Patients with Locally Advanced or Metastatic Solid Tumors (including platinum sensitive ovarian cancer)	PARP	[44]
25	AZD9574 (Alone or in combination with anti-cancer agents)		NCT05417594	Under phase 1/2 trial Trial start: 24 Jun. 2022 Estimated completion: 30 Jun. 2025	Advanced cancer that has recurred/progressed (including ovarian cancer)	PARP	[45]
26	E7449 (Alone or in Combination with Temozolomide (TMZ) or with Carboplatin and Paclitaxel		NCT01618136	Phase 1/2 trial completed in Jul. 2015	Patients with advanced solid tumors (including ovarian cancer)	PARP-1, PARP-2 and tankyrase 1/2	[46,47]
27	NMS-03305293		NCT04182516	Under phase 1 trial Trial start: 25 Nov. 2019 Estimated completion: 30 Dec. 2023	Patients with advanced solid tumors (including ovarian cancer)	PARP	[41]
28	AMXI-5001		ATLAS-101 NCT04503265	Under phase 1/2 trial Trial start: 12 Aug. 2020 Estimated completion: Jan. 2023	Advanced malignant neoplasm (including ovarian cancer) who have failed other therapies	PARP and microtubule polymerization inhibitor	[48]
29	[18F]FluorThanatrace (FTT)		Pilot study	Pilot study	Patients with ovarian carcinoma	PARP-1	[58]

## PARP Inhibitors in Breast and Ovarian Cancers

Olaparib became the first PARP inhibitor to be approved in the clinic (Table 3). Olaparib is being developed for BRCA mutation-positive ovarian cancer [86]. The subsequent phase II studies of Olaparib, in patients with high-grade ovarian cancer revealed a highly statistically significant improvement in progression-free survival (PFS) (median 8.4 months *vs.* 4.8 months; HR, 0.35; *p* < 0.001) [87]. Most interestingly, the patients with a documented germline BRCA mutation showed a significantly higher survival rate (median 11.2 months *vs*. 4.3 months; HR, 0.18; *p* < 0.001) [88]. Subsequent trials established the safety of Olaparib as a single agent, and good responses were witnessed in patients with *BRCA*-mutated breast, ovarian, or prostate tumors [89,90]. Owing to their specificity in targeting cancer cells, only mild side effects have been reported from PARP inhibitor treatment as evident from these trials. Another randomized, double-blind, placebo-controlled phase 3 trial of Olaparib, as a maintenance therapy for BRCA mutated ovarian cancer, SOLO 1 (NCT01844986), led to its approval by the United States Food and Drug Administration (FDA) in 2018. The median progression-free survival was better in the Olaparib treated group (median 49.9 months *vs.* 13.8 months; HR, 0.31; *p* < 0.0001) [91]. The risk of disease progression or death was 70% lower in the Olaparib treated group than the placebo group.

**Table 3:** PARP inhibitors clinically approved by United States FDA (Food and Drug Administration) for metastatic castration-resistant prostate cancer (mCRPC), breast cancer, and ovarian cancer

S.No.	PARP inhibitor	Cancer	Main trial	Status	Cohort	Target	References
1	Rucaparib	Prostate cancer	TRITON2 NCT02952534	Clinically approved, 15 May 2020 (Accelerated approval)	mCRPC with a deleterious BRCA alteration who have received both AR-directed therapy and taxane-based chemotherapy	PARP-1, PARP-2, PARP-3	[59,60]
2	Olaparib		PROfound NCT02987543	Clinically approved, 19 May 2020	mCRPC associated with a deleterious alteration in an HRR gene who received AR- directed therapy	PARP-1, PARP-2, PARP-3	[61–63]
3	Olaparib	Breast cancer	OlympiAD NCT02000622	Clinically approved, 12 Jan. 2018	BRCA mutated HER-2 negative metastatic breast cancer who have received chemotherapy either in neoadjuvant, adjuvant or metastatic setting	PARP-1, PARP-2, PARP-3	[64–68]
			OlympiA NCT02032823	Clinically approved, 11 Mar. 2022	For adjuvant treatment of BRCA mutated HER-2 negative early breast cancer who have received local treatment and neoadjuvant or adjuvant chemotherapy		
4	Talazoparib		EMBRACA NCT01945775	Clinically approved, 16 Oct. 2018	BRCA mutated HER-2 negative locally advanced/ metastatic breast cancer	PARP-1, PARP-2, PARP-16	[64–71]
5	Olaparib	Ovarian cancer	NCT01078662	Clinically approved 19 Dec. 2014	BRCA mutated ovarian cancer who have received 3 or more prior lines of chemotherapy	PARP-1, PARP-2, PARP-3	[72–78]
			SOLO-1 NCT01844986	Clinically approved, 19 Dec. 2018	As first-line maintenance treatment of BRCA-mutated advanced ovarian cancer		
			PAOLA-1 NCT02477644	Clinically approved, 08 May 2020	In combination with bevacizumab for first-line maintenance treatment of homologous recombination deficient (HRD)–positive advanced ovarian cancer		
6	Niraparib		NOVA NCT01847274	Clinically approved, 27 Mar. 2017	Maintenance treatment for platinum sensitive ovarian cancer	PARP-1, PARP-2	[79–83]
			QUADRA NCT02354586	Clinically approved, 23 Oct. 2019	HRD-positive advanced ovarian cancer patients treated with three or more prior chemotherapy regimens		
			PRIMA NCT02655016	Clinically approved, 29 Apr. 2020	Maintenance treatment in patients with advanced Ovarian cancer following complete or partial response to front line platinum-based chemotherapy		
7	Rucaparib		ARIEL3 NCT01968213	Clinically approved, 6 Apr. 2018	Maintenance treatment of recurrent epithelial ovarian, cancer who are sensitive to platinum-based chemotherapy	PARP-1, PARP-2, PARP-3	[84,85]

It is also important to note that BRCA defective cells are much more sensitive to PARP inhibitors than to the knockdown of PARP using a siRNA approach [16]. The study suggested that trapping PARP on specific DNA lesions, may be important for the effective killing of HR-defective cells (Fig. 3). Trapping of PARP leads to stalled replication forks which causes the conversion of single-strand breaks (SSBs) to double-strand breaks (DSBs). These DSBs are repaired by HR-efficient cells, however, in cells deficient in HR, it leads to cell killing. Most PARP inhibitors have almost similar efficacy in inhibiting the catalytic activity of PARP, however, they differ in their ability to trap PARP on the lesions [92]. These studies cemented the foundation for testing of other PARP inhibitors including Rucaparib, Veliparib, and Niraparib in clinical trials for the treatment of breast and ovarian cancer [93–95] (Table 2). The positive findings from subsequent trials in ovarian and breast cancer patients, led FDA to approve two more PARP inhibitors for clinical use in patients with BRCA-mutant ovarian cancer: Rucaparib [96] and Niraparib [97] (Table 3). Along with these, currently, phase 3 trials of Veliparib and Talazoparib are ongoing (NCT02163694 and NCT01945775).

The FDA has also approved two PARP inhibitors for BRCA mutated HER 2 negative breast cancer: Olaparib and talazoparib (BC). Olaparib approval for BRCA mutated HER2 negative metastatic and early breast cancer was based on OlympiAD and OlympiA trials, respectively. OlympiAD was a randomized, open-label, phase 3 trial that compared Olaparib with standard treatment in metastatic breast cancer patients with BRCA mutated HER-2 negative breast cancer [98]. Olaparib treated group showed significantly longer median progression-free survival than the standard therapy group (7.0 months *vs*. 4.2 months; HR, 0.58; *p* < 0.001) and 42% lower risk of disease progression or death than the standard therapy group. Early this year, Olaparib was approved for adjuvant treatment of BRCA-mutated HER-2 negative early breast cancer patients who have received local treatment and neoadjuvant or adjuvant chemotherapy. This approval was based on OlympiA, a randomized double-blind, phase 3 trial. 3-year invasive disease-free survival was better in the Olaparib group (85.9% *vs*. 77.1%; HR, 0.58; *p* < 0.001) and 3-year distant disease-free survival was longer in the Olaparib treated group (87.5% *vs*. 80.4%; HR, 0.57; *p* < 0.001) [65]. Talazoparib was FDA approved for BRCA mutated HER-2 negative locally advanced/metastatic breast cancer based on EMBRACA trial [69,70].

A recent study profiled the crystal structure of the 10 most potent PARP inhibitors by either binding to PARP1 and PARP2 and revealed that veliparib and niraparib are selective inhibitors of PARP1 and PARP2; Olaparib, Rucaparib, and Talazoparib are more potent inhibitors of the PARP1 but are less selective [99] (Fig. 3). Going forward these studies would help improve efficacy and minimize toxicities from PARP inhibitors. Most PARP inhibitors compete with NAD at the active site of PARP-1 enzyme. Since NAD-like PARP inhibitors are less selective as NAD is ubiquitous and NAD competitors could lead to off-target effects, non-NAD-like PARP inhibitors are a novel class of drugs that target histone-dependent activation of PARP-1, a mechanism that is unique to PARP-1. One such inhibitor is 5F02 which showed superior anti-tumor activity in *in vitro* and *in vivo* models of prostate cancer [20].

## PARP Inhibitors-the New Phase of Treatment for Metastatic Prostate Cancer?

The promising outcomes from the clinical trials involving PARP inhibitors for breast and ovarian cancer patients have resulted in a series of studies from research groups across the world to try to broaden the patient cohort which could be benefitted from their use. It is now known that these BRCA mutations also increase the risk of other cancers such as colon (reviewed by Oh et al., [100], melanoma [101], pancreatic, gastric cancers, and prostate cancers (reviewed by Cavanagh et al., [102]). BRCA1 and BRCA2 mutations have shown a pivotal role in DNA repair dysfunction in prostate cancers. Germline BRCA2 carriers have a 5.0 to 8.6-fold increased risk of developing prostate carcinoma [103,104]. Also, the prevalence of germline HR mutations among men with metastatic prostate cancer ranges from 8%–14%, indicative of a large proportion of men with advanced disease may benefit from these agents. In addition, PARP inhibition also showed selective lethality in tumor cells with *TMPRSS2-ERG* gene fusions [105], which are identified in more than 50% of prostate tumors, especially in hormone-insensitive metastatic prostate cancer. Given these findings, as well as the lack of effective treatments for castration-resistant metastatic prostate cancer (mCRPC), PARP inhibitors were tested for efficacy in this patient subset. The phase II open-label, single-arm, two-stage, TOPARP-A trial was conducted to test Olaparib efficacy in tested metastatic-castration resistant prostate cancer (mCRPC) has shown promising results [106]. The study found that 14 out of 16 patients who responded to Olaparib treatment had aberrations in DNA repair-related genes. Further classifying the response based on the mutation showed that all patients with a BRCA2 alteration responded to treatment with Olaparib and defects in ATM were also indicative of response to Olaparib, excluding 1 patient with ATM alteration, who did not respond to therapy. In the rest of 28% patients without DNA repair defects, Olaparib was not observed to be effective [106]. These impressive results led the FDA to grant a breakthrough designation for the use of Olaparib in mCRPC patients with BRCA1/2 or ATM alterations.

The second part of the TOPARP study (TOPARP-B) aimed to validate the role of Olaparib in BRCA2 or ATM carriers and to provide added efficacy data in presence of less common mutations in other DNA repair-related genes, which have been previously linked to PARP inhibitors sensitivity, such as RAD51, FANC, ATR, CDK12, MRE11, CHEK1, CHEK2, and ETS gene fusions. The study confirmed that Olaparib has antitumor activity against heavily pre-treated mCRPC with DDR gene defects, with BRCA1/2 aberrant tumors being the most sensitive but with confirmed responses in patients with other DDR alterations (PALB2 57% (4/7; mPFS 5.3mo); ATM 37% (7/19; mPFS 6.1mo); CDK12 25% (5/20; mPFS 2.9mo)). Another phase 2 trial is also underway to compare abiraterone *vs*. Olaparib as a single agent *vs*. the combination of the two drugs in metastatic CRPC patients with germline or somatic HR mutations.

A phase III PROFOUND trial, in which patients with abiraterone and/or enzalutamide-pretreated CRPC were screened for somatic HR deficiency mutations and then randomized to either AR-targeted therapy or Olaparib. In the Olaparib group, the progression-free survival was significantly longer than the control group (median 7.4 *vs*. 3.6; HR, 0.34; *p* < 0.001). As compared to either enzalutamide or abiraterone, Olaparib was associated with better measures of response. This led to its approval by FDA in May 2020 for mCRPC associated with a deleterious alteration in HRR genes who received AR-directed therapy [61,62].

TRITON 2, a phase 2 trial, led to accelerated approval of rucaparib in May 2020, for mCRPC patients with a deleterious BRCA alteration who have received both AR-directed therapy and taxane-based chemotherapy [59,60]. Another phase 3 trial named TRITON3 is underway to study the efficacy of rucaparib, a potent PARP1, PARP2, and PARP3 inhibitor, in patients with mCRPC associated with HR deficiency (*BRCA1/2* or *ATM* gene mutations) in comparison to *vs*. treatment with physician’s choice of abiraterone acetate, enzalutamide, or docetaxel.

It will be important to see if these trials would validate the use of PARP inhibitors in prostate cancer patients. An integrated genomic analysis of advanced prostate cancer revealed that aberrations of BRCA2, BRCA1, and ATM were observed at substantially higher frequencies (19.3% overall) compared to those in primary prostate cancers [107], which reinforces the testing of PARP inhibitors in mCRPC. Also, considering that most metastatic CRPC patient survival is less than 10 months, PARP inhibitors do open new avenues for mCRPC patients with HR repair deficiency.

## Therapeutic Potential of the Combination of PARP Inhibitors and Immune Checkpoint Inhibitors (ICI)

In the last decade, Immunotherapy has dramatically improved treatment outcomes for cancer patients across multiple tumor types, including lung, melanoma, ovarian, genitourinary, and, more recently, breast cancer, with long-lasting responses. Despite promising results, immunotherapy only benefits a subset of patients due to low overall response rates. There is currently a lot of interest in either in patient selection through biomarkers or finding combinatorial approaches to build synergy with immunotherapy. DNA-damaging agents, in particular, have the potential to improve immunotherapy response by promoting neoantigen release, increasing tumor mutational burden, and increasing PD-L1 expression. The rationale behind using combination of PARP inhibitors in DDR-defective cancer cells is that these prevent single-strand DNA repair, which increases DNA damage, enhancing the load of tumor mutations, thus making the tumors more immunologically “hot” [108]. In DDR-defective cells, trapping of PARP at the lesions was shown to result in stalled replication forks and unrepaired lesions, which increased micronuclei formation. These micronuclei are detected by the cytoplasmic DNA sensor, cGAS, which leads to activation of STING/pTBK1 and type 1 Interferon (IFN) gene signaling that in turn is crucial in mounting robust anti-tumor immune response [108–110]. Several ongoing clinical trials are exploring the benefit of the combination of immunotherapy and PARP inhibition, summarized in Table 4. One such trial, NCT02484404, was carried out for mCRPC, in which the combination of Durvalumab (anti-PDL-1 antibody) and Olaparib was shown to induce PSA responses (reduction ≥ 50%) in 8 out of 17 patients (47%) [110,111]. Progression-free survival was longer in patients with known DDR mutations than the DDR-proficient ones. However, interestingly, the durvalumab and Olaparib combination demonstrated clinical activity in platinum-resistant recurrent ovarian cancer independent of BRCA status. While the data now available indicate that combining PARP inhibitors with ICI could overcome immunological insufficiency, additional data from ongoing trials will be necessary to shed further light on this.

**Table 4:** PARP inhibitors in combination with immunotherapy in various phases of clinical trials for prostate cancer, breast cancer, and ovarian cancer

S.No.	PARP inhibitor	Immunotherapy	Cancer	Main trial	Status	Cohort	References
1	Olaparib	Pembrolizumab (anti-PD-1 antibody)	Prostate cancer	KEYLYNK-010 NCT03834519	Under phase 3 trial Trial start: 02 May 2019 Estimated completion: 29 Sept. 2023	mCRPC	[111–114]
		Durvalumab (anti-PD-L1 antibody) and Cediranib (VEGFR)		NCT04336943	Under phase 2 trial Trial start: 13 Apr. 2021 Estimated completion: 30 Apr. 2025	Prostate cancer with high neoantigen load	
		Durvalumab (anti-PD-L1 antibody)		NCT02484404	Under phase 1/2 trial Trial start: 29 Jun. 2015 Estimated completion: 30 Dec. 2024	Prostate cancer	
		Pembrolizumab (anti-PD-1 antibody)		KEYNOTE-365 NCT02861573	Under phase 1/2 trial Trial start: 17 Nov. 2016 Estimated completion: 30 May 2025	mCRPC	
2	Talazoparib	Avelumab (anti-PD-L1 antibody)		NCT03330405	Under phase 2 trial Trial start: 19 Oct. 2017 Estimated completion: 03 Jan. 2023	castration resistant prostate cancer	[115]
3	Rucaparib	Nivolumab (anti-PD-1 antibody)		CheckMate 9KD NCT03338790	Under phase 2 trial Trial start: 19 Dec. 2017 Estimated completion: 15 Jul. 2023	mCRPC	[116]
4	Pamiparib	BGB-A317 (tislelizumab) (PD-1)		NCT02660034	Phase 1 trial completed on 09 Sep. 2020	mCRPC	[117]
5	Olaparib	Durvalumab (anti-PD-L1 antibody)	Breast cancer	DORA NCT03167619	Phase 2 trial completed on 30 Jun. 2022	Platinum treated triple negative breast cancer	[111,118–122]
		Pembrolizumab (anti-PD-1 antibody)		NCT03025035	Under phase 2 trial Trial start: 10 Sep. 2017 Estimated completion: Nov. 2025	BRCA mutated/ HRD-defect breast cancer	
		Durvalumab (anti-PD-L1 antibody)		NCT02484404	Under phase 1/2 trial Trial start: 29 Jun. 2015 Estimated completion: 30 Dec. 2024	Triple negative breast cancer	
		Atezolizumab (anti-PD-1 antibody)		NCT02849496	Under phase 2 trial Trial start: 15 Nov. 2016 Estimated completion: 31 Aug. 2023	BRCA mutated Non-HER2 positive breast cancer	
		Durvalumab (anti-PD-L1 antibody)		OlympiaN NCT05498155	Under phase 2 trial Trial start: 21 Oct. 2022 Estimated completion: 20 Nov. 2026	BRCA mutated HER2 negative breast cancer	
		Durvalumab (anti-PD-L1 antibody)		NCT03544125	Phase 1 trial completed in 18 Nov. 2020	Metastatic triple negative breast cancer	
6	Talazoparib	Avelumab (anti-PD-L1 antibody)		NCT03330405	Under phase 2 trial Trial start: 19 Oct. 2017 Estimated completion: 03 Jan. 2023	Triple negative breast cancer	[115]
7	Niraparib	Dostarlimab (PD-1) plus radiation therapy		NADiR NCT04837209	Under phase 2 trial Trial start: 21 Jul. 2021 Estimated completion: 01 Dec. 2029	Metastatic triple negative breast cancer	[123–125]
		Pembrolizumab (anti-PD-1 antibody)		TOPACIO NCT02657889	Phase 1 trial completed on 17 Sep. 2021	Triple negative breast cancer	
		TSR-042 (Dostarlimab)		NCT04673448	Under phase 1 trial Trial start: 18 Oct. 2021 Estimated completion: 30 Mar. 2026	Metastatic breast cancer	
8	Pamiparib	BGB-A317 (tislelizumab) (anti-PD-1 antibody)		NCT02660034	Phase 1 trial completed on 09 Sep. 2020	Triple negative breast cancer	[117]
9	Rucaparib	Atezolizumab (anti-PD-1 antibody)		NCT03101280	Phase 1 trial completed on 11 Aug. 2020	Triple negative breast cancer	[126]
10	Olaparib	Durvalumab (anti-PD-L1) and Cediranib (VEGFR)	Ovarian cancer	NCT02484404	Under phase 1/2 trial Trial start: 29 Jun. 2015 Estimated completion: 30 Dec. 2024	Advanced/recurrent ovarian cancer	[111,127–129]
		Tremelimumab (anti-CTLA-4 antibody)		NCT04034927	Under phase 2 trial Trial start: 11 Oct. 2019 Estimated completion: 31 Dec. 2022	Platinum sensitive recurrent ovarian cancer	
		Tremelimumab (anti-CTLA-4 antibody)		NCT02571725	Under phase 1/2 trial Trial start: 23 Feb. 2016 Estimated completion: 15 Jul. 2027	Recurrent BRCA mutated ovarian cancer	
		Durvalumab and tremelimumab		NCT02953457	Under phase 2 trial Trial start: 29 Jun. 2017 Estimated completion: 15 Dec. 2022	BRCA-mutated ovarian cancer	
11	Niraparib	Atezolizumab (anti PD-L1 antibody)		ANITA NCT03598270	Under phase 3 trial Trial start: 21 Nov. 2018 Estimated completion: Jan. 2025	Recurrent ovarian carcinoma	[124,125,130,131]
		Dostarlimab (PD-1)		MOONSTONE NCT03955471	Phase 2 trial completed on 12 Jan. 2022	Platinum resistant ovarian cancer	
		TSR-042 (Dostarlimab)		NCT04673448	Under phase 1 trial Trial start: 18 Oct. 2021 Estimated completion: 30 Mar. 2026	BRCA mutated ovarian cancer	
		Pembrolizumab (anti PD-1 antibody)		NCT02657889	Phase 1 trial completed on 17 Sep. 2021	Recurrent Ovarian cancer	
12	Talazoparib	Avelumab (anti-PD-L1 antibody)		NCT03330405	Under phase 2 trial Trial start: 19 Oct. 2017 Estimated completion: 03 Jan. 2023	recurrent platinum sensitive ovarian cancer	[115]
13	Rucaparib	Atezolizumab (anti-PD-L1 antibody)		NCT03101280	Phase 1 trial completed on 11 Aug. 2020	Advanced ovarian cancer	[126]

## The Road Ahead for PAPR Inhibitors: Promises and Challenges

The clinical trials done with PARP inhibitors so far are highly encouraging and substantiate the fact that these inhibitors could offer better responses not just in breast and ovarian cancer, but in other “PARP-dependent tumors” as well. Data from these clinical trials also argue for the need to change our conventions of treating cancer mainly based on anatomical sites and rather create a “molecular stratification” which may improve treatment response, especially in advanced-stage tumors with limited treatment alternatives. In line with this theory, increasing evidence suggests that BRCA may be inactivated by multiple mechanisms in a large proportion of breast cancers, despite possessing a functional gene structure, a trait now called “BRCAness”. It would be interesting to see whether the use of PARP inhibitors could be extended to these cases as well. On a cautious note, it is also important to draw our attention to breast cancer cases with confirmed BRCA mutations, which do not respond well to PARP inhibitors.

The study 42, which examined Olaparib monotherapy in germline BRCA mutation carriers, found an objective response rate of 34% only [132]. Also, there were cases of ovarian cancers, with no apparent BRCA defect, which responded well to PARP inhibitor therapy. Even though the number of these cases has been marginal, it is important to understand the reason for the failure to improve the predictivity of treatment response. Also, while the distinction in sensitivity between BRCA1 and BRCA2-associated ovarian cancers remains unclear in the clinical setting, emerging *in vitro* data does indicate that all *BRCA* mutations are not equal in their functionality. This suggests that the location of *BRCA1* mutation may influence the efficacy of PARP inhibitors, which should be considered in future studies.

Another important area of active research in the field is identifying accurate biomarkers of response to PARP inhibitors apart from *BRCA* mutation status. Measurement of PARylation levels of peripheral blood mononuclear cells was explored in a study [133]. The homologous recombination deficiency score which has been used to identify patients with defective DNA repair mechanisms (including tumors without *BRCA* mutations) was shown to be associated with increased response rates in neoadjuvant settings [134]. Studies are also undergoing to find methods for evaluation of HR proficiency through the formation of nuclear RAD51 foci [135] as well as the evaluation of BRCA promoter hypermethylation or the levels of 53BP1 expression [136]. However, these results need to be validated in independent large cohorts.

As with other chemotherapeutic drugs, the development of resistance to PARP inhibitors also needs to be addressed. Studies have demonstrated that using PARP inhibitors in cancer cells carrying mutations in *BRCA1* or *BRCA2* could develop resistance by acquiring secondary mutations in the *BRCA* genes that, interestingly, reverse the effect of the original mutation, restoring the levels of functional BRCA proteins [137,138]. Upregulation of genes that encode P-glycoprotein efflux pumps, a known culprit for drug resistance could also hamper the effectiveness of PARP inhibitors [139]. Alterations in signaling pathways have also emerged as mechanisms of PARP1 inhibitor resistance. One example involves the role of microRNAs (miR-622), in modulating the balance of the DNA repair pathway [140]. Another study revealed that phosphorylation of PARP1 at Y907 by c-Met leads to PARP inhibitor resistance and identified c-Met as an important regulator of PAPR inhibitor response [141]. Current approaches to PARP inhibitor resistance are centered on combining PARP inhibition with other DNA damage response inhibitors, immune-checkpoint inhibition, or targeted therapies. To further improve therapeutic outcomes, it is important to improve our understanding of HR-deficient cancers and find agents that target the acquired vulnerabilities of PARP inhibitor-resistant tumors, delay the onset of resistance, or selectively kill unresponsive cells.

## Conclusions

With the advent of techniques like next-generation sequencing, liquid biopsies, and circulating tumor DNA analyses, the possibility of utilizing a personalized therapeutic approach seems highly possible. On the clinical end, ongoing PARP trials would offer significant information on optimal agent selection, scheduling, and dosing either alone or in combination, which will be important for the rationalized use of PARP inhibitors in multiple cancer types. However, overcoming mechanisms of resistance and the identification of reliable predictive biomarkers of response would need to be first addressed using *in vitro* and pre-clinical model systems. With the success of immunotherapy in multiple cancer types, it will also be important to answer if PARP inhibition and immunotherapy could work synergistically and improve overall survival in cancer patients.

## References

[1] Sieber, O. M., Heinimann, K., Tomlinson, I. P. (2003). Genomic instability--the engine of tumorigenesis? Nature Reviews Cancer*,* 3 *(*9*),* 701–708. 10.1038/nrc1170; 12951589

[2] Pors, K., Patterson, L. H. (2005). DNA mismatch repair deficiency, resistance to cancer chemotherapy and the development of hypersensitive agents. Current Topics in Medicinal Chemistry*,* 5 *(*12*),* 1133–1149. 10.2174/156802605774370883; 16248788

[3] Kauffmann, A., Kauffmann, A., Rosselli, F., Lazar, V., Winnepenninckx, V. et al. (2008). High expression of DNA repair pathways is associated with metastasis in melanoma patients. Oncogene*,* 27 *(*5*),* 565–573. 10.1038/sj.onc.1210700; 17891185

[4] Mikhed, Y., Gorlach, A., Knaus, U. G., Daiber, A. (2015). Redox regulation of genome stability by effects on gene expression, epigenetic pathways and DNA damage/repair. Redox Biology*,* 5, 275–289. 10.1016/j.redox.2015.05.008; 26079210 PMC4475862

[5] Bender, A., Pringle, J. R. (1991). Use of a screen for synthetic lethal and multicopy suppressee mutants to identify two new genes involved in morphogenesis in *Saccharomyces cerevisiae*. Molecular and Cellular Biology*,* 11 *(*3*),* 1295–1305. 10.1128/mcb.11.3.1295-1305.1991; 1996092 PMC369400

[6] Kaelin Jr, W. G. (2005). The concept of synthetic lethality in the context of anticancer therapy. Nature Reviews Cancer*,* 5 *(*9*),* 689–698. 10.1038/nrc1691; 16110319

[7] McLornan, D. P., List, A., Mufti, G. J. (2014). Applying synthetic lethality for the selective targeting of cancer. The New England Journal of Medicine*,* 371 *(*18*),* 1725–1735. 10.1056/NEJMra1407390; 25354106

[8] Ame, J. C., Spenlehauer, C., de Murcia, G. (2004). The PARP superfamily. BioEssays*,* 26 *(*8*),* 882–893. 10.1002/bies.20085; 15273990

[9] Caldecott, K. W., Aoufouchi, S., Johnson, P., Shall, S. (1996). XRCC1 polypeptide interacts with DNA polymerase beta and possibly poly (ADP-ribose) polymerase, and DNA ligase III is a novel molecular ‘nick-sensor’ *in vitro*. Nucleic Acids Research*,* 24 *(*22*),* 4387–4394. 10.1093/nar/24.22.4387; 8948628 PMC146288

[10] Haince, J. F., McDonald, D., Rodrigue, A., Déry, U., Masson, J. Y. et al. (2008). PARP1-dependent kinetics of recruitment of MRE11 and NBS1 proteins to multiple DNA damage sites. The Journal of Biological Chemistry*,* 283 *(*2*),* 1197–1208. 10.1074/jbc.M706734200; 18025084

[11] de Murcia, J. M., Niedergang, C., Trucco, C., Ricoul, M., Dutrillaux, B. et al. (1997). Requirement of poly(ADP-ribose) polymerase in recovery from DNA damage in mice and in cells. Proceedings of the National Academy of Sciences*,* 94 *(*14*),* 7303–7307. 10.1073/pnas.94.14.7303; 9207086 PMC23816

[12] Masutani, M., Nozaki, T., Nakamoto, K., Nakagama, H., Suzuki, H. et al. (2000). The response of Parp knockout mice against DNA damaging agents. Mutational Research*,* 462 *(*2–3*),* 159–166. 10.1016/S1383-5742(00)00033-8; 10767627

[13] Calabrese, C. R., Almassy, R., Barton, S., Batey, M. A., Calvert, A. H. et al. (2004). Anticancer chemosensitization and radiosensitization by the novel poly(ADP-ribose) polymerase-1 inhibitor AG14361. Journal of National Cancer Institute*,* 96 *(*1*),* 56–67. 10.1093/jnci/djh005; 14709739

[14] Miknyoczki, S. J., Jones-Bolin, S., Pritchard, S., Hunter, K., Zhao, H. et al. (2003). Chemopotentiation of temozolomide, irinotecan, and cisplatin activity by CEP-6800, a poly(ADP-ribose) polymerase inhibitor. Molecular Cancer Therapeutics*,* 2 *(*4*),* 371–382. 10.1158/1535-7163.MCT-07-0062 12700281

[15] Chalmers, A., Johnston, P., Woodcock, M., Joiner, M., Marples, B. (2004). PARP-1, PARP-2, and the cellular response to low doses of ionizing radiation. International Journal of Radiation Oncology Biology Physics*,* 58 *(*2*),* 410–419. 10.1016/j.ijrobp.2003.09.053; 14751510

[16] Bryant, H. E., Schultz, N., Thomas, H. D., Parker, K. M., Flower, D. et al. (2005). Specific killing of BRCA2-deficient tumours with inhibitors of poly(ADP-ribose) polymerase. Nature*,* 434 *(*7035*),* 913–917. 10.1038/nature03443; 15829966

[17] Farmer, H., McCabe, N., Lord, C. J., Tutt, A. N., Johnson, D. A. et al. (2005). Targeting the DNA repair defect in BRCA mutant cells as a therapeutic strategy. Nature*,* 434 *(*7035*),* 917–921. 10.1038/nature03445; 15829967

[18] Jasin, M. (2002). Homologous repair of DNA damage and tumorigenesis: The BRCA connection. Oncogene*,* 21 *(*58*),* 8981–8993. 10.1038/sj.onc.1206176; 12483514

[19] Mavaddat, N., Peock, S., Frost, D., Ellis, S., Platte, R. et al. (2013). Cancer risks for BRCA1 and BRCA2 mutation carriers: Results from prospective analysis of EMBRACE. Journal of the National Cancer Institute*,* 105 *(*11*),* 812–822. 10.1093/jnci/djt095; 23628597

[20] Karpova, Y., Wu, C., Divan, A., McDonnell, M. E., Hewlett, E. et al. (2019). Non-NAD-like PARP-1 inhibitors in prostate cancer treatment. Biochemical Pharmacology*,* 167 *(*1*),* 149–162. 10.1016/j.bcp.2019.03.021; 30880062 PMC6702078

[21] Yuan, B., Ye, N., Song, S. S., Wang, Y. T., Song, Z. et al. (2017). Poly(ADP-ribose) polymerase (PARP) inhibition and anticancer activity of simmiparib, a new inhibitor undergoing clinical trials. Cancer Letters*,* 386*,* 47–56. 10.1016/j.canlet.2016.11.010; 27847302

[22] Wang, L., Zhang, S., Yu, X., Guo, C. (2020). Novel poly (ADP-ribose) polymerase-1 inhibitor DDHCB inhibits proliferation of BRCA mutant breast cancer cell *in vitro* and *in vivo* through a synthetic lethal mechanism. Chemical Research in Toxicology*,* 33 *(*7*),* 1874–1881. 10.1021/acs.chemrestox.0c00087; 32394702

[23] Guo, C., Zhang, F., Wu, X., Yu, X., Wu, X. et al. (2020). BTH-8, a novel poly (ADP-ribose) polymerase-1 (PARP-1) inhibitor, causes DNA double-strand breaks and exhibits anticancer activities *in vitro* and *in vivo*. International Journal of Biological Macromolecules*,* 150*,* 238–245. 10.1016/j.ijbiomac.2020.02.069; 32057845

[24] Du, T., Zhang, Z., Zhou, J., Sheng, L., Yao, H. et al. (2022). A novel PARP inhibitor YHP-836 for the treatment of BRCA-deficiency cancers. Frontiers in Pharmacology*,* 13*,* 22. 10.3389/fphar.2022.865085; 35910366 PMC9326368

[25] Tian, C., Wei, Y., Li, J., Huang, Z., Wang, Q. et al. (2022). A novel CDK4/6 and PARP dual inhibitor ZC-22 effectively suppresses tumor growth and improves the response to cisplatin treatment in breast and ovarian cancer. International Journal of Molecular Sciences*,* 23 *(*5*),* 2892. 10.3390/ijms23052892; 35270034 PMC8911181

[26] He, J. X., Wang, M., Huan, X. J., Chen, C. H., Song, S. S. et al. (2017). Novel PARP1/2 inhibitor mefuparib hydrochloride elicits potent *in vitro* and *in vivo* anticancer activity, characteristic of high tissue distribution. Oncotarget*,* 8 *(*3*),* 4156–4168. 10.18632/oncotarget.13749; 27926532 PMC5354820

[27] Boraei, A. T., Singh, P. K., Sechi, M., Satta, S. (2019). Discovery of novel functionalized 1, 2, 4-triazoles as PARP-1 inhibitors in breast cancer: Design, synthesis and antitumor activity evaluation. European Journal of Medicinal Chemistry*,* 182*,* 111621. 10.1016/j.ejmech.2019.111621; 31442685

[28] Putri, J. F., Bhargava, P., Dhanjal, J. K., Yaguchi, T., Sundar, D. et al. (2019). Mortaparib, a novel dual inhibitor of mortalin and PARP1, is a potential drug candidate for ovarian and cervical cancers. Journal of Experimental & Clinical Cancer Research*,* 38 *(*1*),* 1–15. 10.1186/s13046-019-1500-9; 31856867 PMC6923857

[29] Ghafouri-Fard, S., Khoshbakht, T., Hussen, B. M., Dong, P., Gassler, N. et al. (2022). A review on the role of cyclin dependent kinases in cancers. Cancer Cell International*,* 22 *(*1*),* 1–69. 10.1186/s12935-022-02747-z; 36266723 PMC9583502

[30] Smith, M. R., Scher, H. I., Sandhu, S., Efstathiou, E., Lara, P. N. et al. (2022). Niraparib in patients with metastatic castration-resistant prostate cancer and DNA repair gene defects (GALAHAD): A multicentre, open-label, phase 2 trial. The Lancet Oncology*,* 23 *(*3*),* 362–373. 10.1016/S1470-2045(21)00757-9; 35131040 PMC9361481

[31] ClinicalTrials.gov U.S (2016). National library of medicine identifier NCT02854436 an efficacy and safety study of niraparib in men with metastatic castration-resistant prostate cancer and DNA-repair anomalies. https://clinicaltrials.gov/ct2/show/NCT02854436

[32] de Bono, J. S., Mehra, N., Scagliotti, G. V., Castro, E., Dorff, T. et al. (2021). Talazoparib monotherapy in metastatic castration-resistant prostate cancer with DNA repair alterations (TALAPRO-1): An open-label, phase 2 trial. The Lancet Oncology*,* 22 *(*9*),* 1250–1264. 10.1016/S1470-2045(21)00376-4; 34388386

[33] ClinicalTrials.gov U.S (2017). National library of medicine identifier NCT03148795 a study of talazoparib in men with DNA repair defects and metastatic castration-resistant prostate cancer. https://clinicaltrials.gov/ct2/show/NCT03148795

[34] ClinicalTrials.gov U.S (2022). National library of medicine identifier NCT05327621 Pamiparib in mCRPC with HRD or BRCA1/2 mutation. https://clinicaltrials.gov/ct2/show/NCT05327621

[35] ClinicalTrials.gov U.S (2012). National library of medicine identifier NCT01576172 abiraterone acetate and prednisone with or without veliparib in treating patients with metastatic castration-resistant prostate cancer. https://clinicaltrials.gov/ct2/show/NCT01576172

[36] ClinicalTrials.gov U.S (2020). National library of medicine identifier NCT04296370 a study of fluzoparib±Apatinib versus chemotherapy treatment of physician’s choice in HER2-negative metastatic breast cancer patients with germline BRCA mutation. https://clinicaltrials.gov/ct2/show/NCT04296370

[37] ClinicalTrials.gov U.S (2022). National library of medicine identifier NCT05232006 PARP-inhibitor on advanced metastatic breast cancer in germline PALB2 mutations carriers. https://clinicaltrials.gov/ct2/show/NCT05232006

[38] ClinicalTrials.gov U.S (2014). National library of medicine identifier NCT01905592 a phase III trial of niraparib versus physician’s choice in HER2 negative, germline BRCA mutation-positive breast cancer patients. https://clinicaltrials.gov/ct2/show/NCT01905592

[39] ClinicalTrials.gov U.S (2018). National library of medicine identifier NCT03562832 investigation of anti-tumour effect and tolerability of the PARP inhibitor 2X-121 in patients with metastatic breast cancer selected by the 2X-121 DRP. https://clinicaltrials.gov/ct2/show/NCT03562832

[40] ClinicalTrials.gov U.S (2016). National library of medicine identifier NCT02505048 a study to assess the efficacy of rucaparib in metastatic breast cancer patients with a BRCAness genomic signature. https://clinicaltrials.gov/ct2/show/NCT02505048

[41] ClinicalTrials.gov U.S (2019). National library of medicine identifier NCT04182516 study of NMS-03305293 in pts with selected advanced/metastatic solid tumors. https://clinicaltrials.gov/ct2/show/NCT04182516

[42] Illuzzi, G., Staniszewska, A. D., Gill, S. J., Pike, A., McWilliams, L. et al. (2022). Preclinical characterization of AZD5305, a next generation, highly selective PARP1 inhibitor and trapper. Clinical Cancer Research*,* 28 *(*21*),* 4724–4736. 10.1158/1078-0432.CCR-22-0301; 35929986 PMC9623235

[43] ClinicalTrials.gov U.S (2020). National library of medicine identifier NCT04644068 study of AZD5305 as monotherapy and in combination with anti-cancer agents in patients with advanced solid malignancies. https://clinicaltrials.gov/ct2/show/NCT04644068

[44] ClinicalTrials.gov U.S (2021). National library of medicine identifier NCT05002868 safety, pharmacokinetics and anti-tumor activity of RP12146, a PARP inhibitor, in patients with locally advanced or metastatic solid tumors. https://clinicaltrials.gov/ct2/show/NCT05002868

[45] ClinicalTrials.gov U.S (2022). National library of medicine identifier NCT05417594 study of AZD9574 as monotherapy and in combination with anti-cancer agents in participants with advanced solid malignancies. https://clinicaltrials.gov/ct2/show/NCT05417594

[46] ClinicalTrials.gov U.S (2012). National library of medicine identifier NCT01618136 an open-label, multicenter, phase 1/2 study of Poly(ADP-Ribose) polymerase (PARP) inhibitor E7449 as single agent in subjects with advanced solid tumors or with B-cell malignancies and in combination with temozolomide (TMZ) or with carboplatin and paclitaxel in subjects with advanced solid tumors. https://clinicaltrials.gov/ct2/show/NCT01618136

[47] Plummer, R., Dua, D., Cresti, N., Drew, Y., Stephens, P. et al. (2020). First-in-human study of the PARP/tankyrase inhibitor E7449 in patients with advanced solid tumours and evaluation of a novel drug-response predictor. British Journal of Cancer*,* 123 *(*4*),* 525–533. 10.1038/s41416-020-0916-5; 32523090 PMC7434893

[48] ClinicalTrials.gov U.S (2020). National library of medicine identifier NCT0450326 a trial of AMXI-5001 for treatment in patients with advanced malignancies. https://clinicaltrials.gov/ct2/show/NCT04503265

[49] ClinicalTrials.gov U.S (2016). National library of medicine identifier NCT03333915 study of the efficacy, safety and pharmacokinetics of pamiparib (BGB-290) in participants with advanced solid tumors. https://clinicaltrials.gov/ct2/show/NCT03333915

[50] Yuan, B., Ye, N., Song, S. S., Wang, Y. T., Song, Z. et al. (2017). Poly (ADP-ribose) polymerase (PARP) inhibition and anticancer activity of simmiparib, a new inhibitor undergoing clinical trials. Cancer Letters*,* 386*,* 47–56. 10.1016/j.canlet.2016.11.010; 27847302

[51] ClinicalTrials.gov U.S (2016). National library of medicine identifier NCT02993913 safety, tolerability and pharmacokinetics of simmiparib in patients with malignant advanced solid tumor. https://www.clinicaltrials.gov/ct2/show/NCT02993913

[52] ClinicalTrials.gov U.S (2015). National library of medicine identifier NCT02470585 veliparib with carboplatin and paclitaxel and as continuation maintenance therapy in adults with newly diagnosed stage III or IV, high-grade serous, epithelial ovarian, fallopian tube, or primary peritoneal cancer. https://clinicaltrials.gov/ct2/show/NCT02470585

[53] ClinicalTrials.gov U.S (2019). National library of medicine identifier NCT04169997 a study of IMP4297 as maintenance treatment following first-line chemotherapy in patients with advanced ovarian cancer. https://clinicaltrials.gov/ct2/show/NCT04169997

[54] ClinicalTrials.gov U.S (2021). National library of medicine identifier NCT0459832 BrUOG 390: Neoadjuvant treatment with talazoparib. https://www.clinicaltrials.gov/ct2/show/NCT04598321

[55] ClinicalTrials.gov U.S (2022). National library of medicine identifier NCT05489926 a study to explore pamiparib treatment in epithelial ovarian cancer after prior PARP inhibitor exposure. https://www.clinicaltrials.gov/ct2/show/NCT05489926

[56] ClinicalTrials.gov U.S (2018). National library of medicine identifier NCT03509636 a study of fluzoparib(SHR-3162)in BRCA1/2-mutant relapsed ovarian cancer. https://clinicaltrials.gov/ct2/show/NCT03509636

[57] Li, N., Bu, H., Liu, J., Zhu, J., Zhou, Q. et al. (2021). An open-label, multicenter, single-arm, phase II study of fluzoparib in patients with germline *BRCA1/2* mutation and platinum-sensitive recurrent ovarian cancer. Clinical Cancer Research*,* 27 *(*9*),* 2452–2458. 10.1158/1078-0432.CCR-20-3546; 33558426

[58] Pantel, A., MaKvandi, M., Doot, R., Schwartz, L., Greenberg, R. et al. (2017). A pilot study of a novel poly (ADP-ribose) polymerase-1 (PARP) PET tracer ([18F] FluorThanatrace) in patients with ovarian carcinoma. Journal of Nuclear Medicine*,* 58 *(*S1*),* 386.

[59] FDA grants accelerated approval to rucaparib for BRCA-mutated metastatic castration-resistant prostate cancer (2020). Food and Drug Administration. https://www.fda.gov/drugs/resources-information-approved-drugs/fda-grants-accelerated-approval-rucaparib-brca-mutated-metastatic-castration-resistant-prostate

[60] ClinicalTrials.gov U.S (2017). National library of medicine identifier NCT02952534 a study of rucaparib in patients with metastatic castration-resistant prostate cancer and homologous recombination gene deficiency. https://clinicaltrials.gov/ct2/show/NCT02952534

[61] FDA approves Olaparib for HRR gene-mutated metastatic castration-resistant prostate cancer (2020). Food and Drug Administration. https://www.fda.gov/drugs/resources-information-approved-drugs/fda-approves-Olaparib-hrr-gene-mutated-metastatic-castration-resistant-prostate-cancer

[62] de Bono, J., Mateo, J., Fizazi, K., Saad, F., Shore, N. et al. (2020). Olaparib for metastatic castration-resistant prostate cancer. New England Journal of Medicine*,* 382 *(*22*),* 2091–2102. 10.1056/NEJMoa1911440; 32343890

[63] ClinicalTrials.gov U.S (2017). National library of medicine identifier NCT02987543 study of olaparib (Lynparza™) versus enzalutamide or abiraterone acetate in men with metastatic castration-resistant prostate cancer (PROfound Study). https://clinicaltrials.gov/ct2/show/NCT02987543

[64] Kumar, M., Jaiswal, R. K., Yadava, P. K., Singh, R. P. (2020). An assessment of poly (ADP-ribose) polymerase-1 role in normal and cancer cells. BioFactors*,* 46 *(*6*),* 894–905. 10.1002/biof.1688; 33098603

[65] Tutt, A. N., Garber, J. E., Kaufman, B., Viale, G., Fumagalli, D. et al. (2021). Adjuvant Olaparib for patients with BRCA1-or BRCA2-mutated breast cancer. New England Journal of Medicine*,* 384 *(*25*),* 2394–2405. 10.1056/NEJMoa2105215; 34081848 PMC9126186

[66] FDA approves Olaparib for germline BRCA-mutated metastatic breast cancer (2018). Food and Drug Administration. https://www.fda.gov/drugs/resources-information-approved-drugs/fda-approves-Olaparib-germline-brca-mutated-metastatic-breast-cancer

[67] FDA approves Olaparib for adjuvant treatment of high-risk early breast cancer (2022). Food and Drug Administration. https://www.fda.gov/drugs/resources-information-approved-drugs/fda-approves-Olaparib-adjuvant-treatment-high-risk-early-breast-cancer

[68] Tung, N., Garber, J. E. (2022). PARP inhibition in breast cancer: Progress made and future hopes. Nature Partner Journals Breast Cancer*,* 8 *(*1*),* 1–5. 10.1038/s41523-022-00411-3; 35396508 PMC8993852

[69] FDA approves talazoparib for gBRCAm HER2-negative locally advanced or metastatic breast cancer (2018). Food and Drug Administration. https://www.fda.gov/drugs/drug-approvals-and-databases/fda-approves-talazoparib-gbrcam-her2-negative-locally-advanced-or-metastatic-breast-cancer

[70] Litton, J. K., Rugo, H. S., Ettl, J., Hurvitz, S. A., Gonçalves, A. et al. (2018). Talazoparib in patients with advanced breast cancer and a germline BRCA mutation. New England Journal of Medicine*,* 379 *(*8*),* 753–763. 10.1056/NEJMoa1802905; 30110579 PMC10600918

[71] ClinicalTrials.gov U.S (2013). National library of medicine identifier NCT01945775 a study evaluating talazoparib (BMN 673), a PARP inhibitor, in advanced and/or metastatic breast cancer patients with BRCA mutation (EMBRACA Study). https://clinicaltrials.gov/ct2/show/NCT01945775

[72] ClinicalTrials.gov U.S (2013). National library of medicine identifier NCT01844986 olaparib maintenance monotherapy in patients with brca mutated ovarian cancer following first line platinum based chemotherapy. https://clinicaltrials.gov/ct2/show/NCT01844986

[73] ClinicalTrials.gov U.S (2015). National library of medicine identifier NCT02477644 platine, avastin and olaparib in 1st line. https://clinicaltrials.gov/ct2/show/NCT02477644

[74] Arora, S., Balasubramaniam, S., Zhang, H., Berman, T., Narayan, P. et al. (2021). FDA approval summary: Olaparib monotherapy or in combination with bevacizumab for the maintenance treatment of patients with advanced ovarian cancer. The Oncologist*,* 26 *(*1*),* e164–e172. 10.1002/onco.13551; 33017510 PMC7794199

[75] Klotz, D. M., Wimberger, P. (2020). Overcoming PARP inhibitor resistance in ovarian cancer: What are the most promising strategies? Archives of Gynecology and Obstetrics*,* 302 *(*5*),* 1087–1102. 10.1007/s00404-020-05677-1; 32833070 PMC7524817

[76] Kim, G., Ison, G., McKee, A. E., Zhang, H., Tang, S. et al. (2015). FDA approval summary: Olaparib monotherapy in patients with deleterious germline *BRCA*-mutated advanced ovarian cancer treated with three or more lines of Chemotherapy. Clinical Cancer Research*,* 21 *(*19*),* 4257–4261. 10.1158/1078-0432.CCR-15-0887; 26187614

[77] Kaufman, B., Shapira-Frommer, R., Schmutzler, R. K., Audeh, M. W., Friedlander, M. et al. (2015). Olaparib monotherapy in patients with advanced cancer and a germline BRCA1/2 mutation. Journal of Clinical Oncology*,* 33 *(*3*),* 244–250. 10.1200/JCO.2014.56.2728; 25366685 PMC6057749

[78] Ray-Coquard, I., Pautier, P., Pignata, S., Pérol, D., González-Martín, A. et al. (2019). Olaparib plus bevacizumab as first-line maintenance in ovarian cancer. New England Journal of Medicine*,* 381 *(*25*),* 2416–2428. 10.1056/NEJMoa1911361; 31851799

[79] FDA approves niraparib for first-line maintenance of advanced ovarian cancer (2020). Food and Drug Administration. https://www.fda.gov/drugs/resources-information-approved-drugs/fda-approves-niraparib-first-line-maintenance-advanced-ovarian-cancer

[80] ClinicalTrials.gov U.S (2016). National library of medicine identifier NCT02655016 a study of niraparib (GSK3985771) maintenance treatment in participants with advanced ovarian cancer following response on front-line platinum-based chemotherapy. https://clinicaltrials.gov/ct2/show/NCT02655016

[81] FDA approves niraparib for HRD-positive advanced ovarian cancer (2019). Food and Drug Administration. https://www.fda.gov/drugs/resources-information-approved-drugs/fda-approves-niraparib-hrd-positive-advanced-ovarian-cancer

[82] ClinicalTrials.gov U.S (2015). National library of medicine identifier NCT02354586 a study of niraparib in patients with ovarian cancer who have received three or four previous chemotherapy regimens. https://clinicaltrials.gov/ct2/show/NCT02354586

[83] Niraparib (ZEJULA) | FDA (2017). Food and Drug Administration. https://www.fda.gov/drugs/resources-information-approved-drugs/niraparib-zejula

[84] FDA approves rucaparib for maintenance treatment of recurrent ovarian, fallopian tube, or primary peritoneal cancer (2018). Food and Drug Administration. https://www.fda.gov/drugs/resources-information-approved-drugs/fda-approves-rucaparib-maintenance-treatment-recurrent-ovarian-fallopian-tube-or-primary-peritoneal 10.1097/01.NAJ.0000544162.67563.96 30048284

[85] ClinicalTrials.gov U.S (2014). National library of medicine identifier NCT01968213 a study of rucaparib as switch maintenance following platinum-based chemotherapy in patients with platinum-sensitive, high-grade serous or endometrioid epithelial ovarian, primary peritoneal or fallopian tube cancer. https://clinicaltrials.gov/ct2/show/NCT01968213

[86] Deeks, E. D. (2015). Olaparib: First global approval. Drugs*,* 75 *(*2*),* 231–240. 10.1007/s40265-015-0345-6; 25616434

[87] Ledermann, J., Harter, P., Gourley, C., Friedlander, M., Vergote, I. et al. (2012). Olaparib maintenance therapy in platinum-sensitive relapsed ovarian cancer. New England Journal of Medicine*,* 366 *(*15*),* 1382–1392. 10.1056/NEJMoa1105535; 22452356

[88] Ledermann, J., Harter, P., Gourley, C., Friedlander, M., Vergote, I. et al. (2014). Olaparib maintenance therapy in patients with platinum-sensitive relapsed serous ovarian cancer: A preplanned retrospective analysis of outcomes by BRCA status in a randomised phase 2 trial. Lancet Oncology*,* 15 *(*8*),* 852–861. 10.1016/S1470-2045(14)70228-1; 24882434

[89] Audeh, M. W., Carmichael, J., Penson, R. T., Friedlander, M., Powell, B. et al. (2010). Oral poly(ADP-ribose) polymerase inhibitor Olaparib in patients with BRCA1 or BRCA2 mutations and recurrent ovarian cancer: A proof-of-concept trial. Lancet*,* 376 *(*9737*),* 245–251. 10.1016/S0140-6736(10)60893-8; 20609468

[90] Tutt, A., Robson, M., Garber, J. E., Domchek, S. M., Audeh, M. W. et al. (2010). Oral poly(ADP-ribose) polymerase inhibitor Olaparib in patients with BRCA1 or BRCA2 mutations and advanced breast cancer: A proof-of-concept trial. Lancet*,* 376*,* 235–244. 10.1016/S0140-6736(10)60893-8; 20609467

[91] Moore, K., Colombo, N., Scambia, G., Kim, B. G., Oaknin, A. et al. (2018). Maintenance Olaparib in patients with newly diagnosed advanced ovarian cancer. New England Journal of Medicine*,* 379 *(*26*),* 2495–2505. 10.1056/NEJMoa1810858; 30345884

[92] Murai, J., Huang, S. Y. N., Das, B. B., Renaud, A., Zhang, Y. et al. (2012). Trapping of PARP1 and PARP2 by clinical PARP inhibitors. Cancer Research*,* 72 *(*21*),* 5588–5599. 10.1158/0008-5472.CAN-12-2753; 23118055 PMC3528345

[93] Dockery, L. E., Gunderson, C. C., Moore, K. N. (2017). Rucaparib: The past, present, and future of a newly approved PARP inhibitor for ovarian cancer. Onco Targets and Therapy*,* 10*,* 3029–3037. 10.2147/OTT.S114714; 28790837 PMC5488752

[94] Coleman, R. L., Sill, M. W., Bell-McGuinn, K., Aghajanian, C., Gray, H. J. et al. (2015). A phase II evaluation of the potent, highly selective PARP inhibitor veliparib in the treatment of persistent or recurrent epithelial ovarian, fallopian tube, or primary peritoneal cancer in patients who carry a germline BRCA1 or BRCA2 mutation-An NRG Oncology/Gynecologic Oncology Group study. Gynecologic Oncology*,* 137 *(*3*),* 386–391. 10.1016/j.ygyno.2015.03.042; 25818403 PMC4447525

[95] Heo, Y. A., Duggan, S. T. (2018). Niraparib: A review in ovarian cancer. Targeted Oncology*,* 13 *(*4*),* 533–539. 10.1007/s11523-018-0582-1; 30073633

[96] Balasubramaniam, S., Beaver, J. A., Horton, S., Fernandes, L. L., Tang, S. et al. (2017). FDA approval summary: Rucaparib for the treatment of patients with deleterious BRCA mutation-associated advanced ovarian cancer. Clinical Cancer Research*,* 23 *(*23*),* 7165–7170. 10.1158/1078-0432.CCR-17-1337; 28751443

[97] Ison, G., Howie, L. J., Amiri-Kordestani, L., Zhang, L., Tang, S. et al. (2018). FDA approval summary: Niraparib for the maintenance treatment of patients with recurrent ovarian cancer in response to platinum-based chemotherapy. Clinical Cancer Research*,* 24 *(*17*),* 4066–4071. 10.1158/1078-0432.CCR-18-0042; 29650751

[98] Robson, M., Im, S. A., Senkus, E., Xu, B., Domchek, S. M. et al. (2017). Olaparib for metastatic breast cancer in patients with a germline BRCA mutation. New England Journal of Medicine*,* 377 *(*6*),* 523–533. 10.1056/NEJMoa1706450; 28578601

[99] Thorsell, A. G., Ekblad, T., Karlberg, T., Löw, M., Pinto, A. F. et al. (2017). Structural basis for potency and promiscuity in Poly(ADP-ribose) polymerase (PARP) and tankyrase inhibitors. Journal of Medicinal Chemistry*,* 60 *(*4*),* 1262–1271. 10.1021/acs.jmedchem.6b00990; 28001384 PMC5934274

[100] Oh, M., McBride, A., Yun, S., Bhattacharjee, S., Slack, M. et al. (2018). *BRCA1* and *BRCA2* gene mutations and colorectal cancer risk: Systematic review and meta-analysis. Journal of National Cancer Institute*,* 110 *(*11*),* 1178–1189. 10.1093/jnci/djy148; 30380096

[101] Gumaste, P. V., Penn, L. A., Cymerman, R. M., Kirchhoff, T., Polsky, D. et al. (2015). Skin cancer risk in BRCA1/2 mutation carriers. British Journal of Dermatology*,* 172 *(*6*),* 1498–1506. 10.1111/bjd.13626; 25524463 PMC5785081

[102] Cavanagh, H., Rogers, K. M. (2015). The role of *BRCA1* and *BRCA2* mutations in prostate, pancreatic and stomach cancers. Hereditary Cancer in Clinical Practice*,* 13 *(*1*),* 16. 10.1186/s13053-015-0038-x; 26236408 PMC4521499

[103] Castro, E., Goh, C., Olmos, D., Saunders, E., Leongamornlert, D. et al. (2013). Germline BRCA mutations are associated with higher risk of nodal involvement, distant metastasis, and poor survival outcomes in prostate cancer. Journal of Clinical Oncology*,* 31 *(*14*),* 1748–1757. 10.1200/JCO.2012.43.1882; 23569316 PMC3641696

[104] Bancroft, E. K., Page, E. C., Castro, E., Lilja, H., Vickers, A. et al. (2014). Targeted prostate cancer screening in BRCA1 and BRCA2 mutation carriers: Results from the initial screening round of the IMPACT study. European Urology*,* 66 *(*3*),* 489–499. 10.1016/j.eururo.2014.01.003; 24484606 PMC4105321

[105] Chatterjee, P., Choudhary, G. S., Alswillah, T., Xiong, X., Heston, W. D. et al. (2015). The TMPRSS2-ERG gene fusion blocks XRCC4-mediated nonhomologous end-joining repair and radiosensitizes prostate cancer cells to PARP inhibition. Molecular Cancer Therapeutics*,* 14 *(*8*),* 1896–1906. 10.1158/1535-7163.MCT-14-0865; 26026052 PMC4529796

[106] Mateo, J., Carreira, S., Sandhu, S., Miranda, S., Mossop, H. et al. (2015). DNA-repair defects and olaparib in metastatic prostate cancer. New England Journal of Medicine*,* 373 *(*18*),* 1697–1708. 10.1056/NEJMoa1506859; 26510020 PMC5228595

[107] Robinson, D., van Allen, E. M., Wu, Y. M., Schultz, N., Lonigro, R. J. et al. (2015). Integrative clinical genomics of advanced prostate cancer. Cell*,* 162 *(*5*),* 454. 10.1016/j.cell.2015.05.001; 28843286

[108] Chabanon, R. M., Muirhead, G., Krastev, D. B., Adam, J., Morel, D. et al. (2019). PARP inhibition enhances tumor cell-intrinsic immunity in ERCC1-deficient non-small cell lung cancer. The Journal of Clinical Investigation*,* 129 *(*3*),* 1211–1228. 10.1172/JCI123319; 30589644 PMC6391116

[109] Teyssonneau, D., Margot, H., Cabart, M., Anonnay, M., Sargos, P. et al. (2015). Prostate cancer and PARP inhibitors: Progress and challenges. Journal of Hematology & Oncology*,* 14 *(*1*),* 1–19. 10.1186/s13045-021-01061-x; 33781305 PMC8008655

[110] Peyraud, F., Italiano, A. (2020). Combined PARP inhibition and immune checkpoint therapy in solid tumors. Cancers*,* 12 *(*6*),* 1502. 10.3390/cancers12061502; 32526888 PMC7352466

[111] ClinicalTrials.gov U.S (2015). National library of medicine identifier NCT02484404 Phase I/II study of the anti-programmed death ligand-1 durvalumab antibody (MEDI4736) in combination with olaparib and/or cediranib for advanced solid tumors and advanced or recurrent ovarian, triple negative breast, lung, prostate and colorectal can. https://clinicaltrials.gov/ct2/show/NCT02484404

[112] ClinicalTrials.gov U.S (2016). National library of medicine identifier NCT0286157 study of pembrolizumab (MK-3475) combination therapies in metastatic castration-resistant prostate cancer (MK-3475-365/KEYNOTE-365). https://clinicaltrials.gov/ct2/show/NCT02861573

[113] ClinicalTrials.gov U.S (2019). National library of medicine identifier NCT03834519 study of pembrolizumab (MK-3475) plus olaparib versus abiraterone acetate or enzalutamide in metastatic castration-resistant prostate cancer (mCRPC) (MK-7339-010/KEYLYNK-010). https://clinicaltrials.gov/ct2/show/NCT03834519

[114] ClinicalTrials.gov U.S (2021). National library of medicine identifier NCT04336943 durvalumab and olaparib for the treatment of prostate cancer in men predicted to have a high neoantigen load. https://clinicaltrials.gov/ct2/show/NCT04336943

[115] ClinicalTrials.gov U.S (2017). National library of medicine identifier NCT03330405 javelin parp medley: Avelumab plus talazoparib in locally advanced or metastatic solid tumors. https://clinicaltrials.gov/ct2/show/NCT03330405

[116] ClinicalTrials.gov U.S (2017). National library of medicine identifier NCT03338790 an investigational immunotherapy study of nivolumab in combination with rucaparib, docetaxel, or enzalutamide in metastatic castration-resistant prostate cancer (CheckMate 9KD). https://clinicaltrials.gov/ct2/show/NCT03338790

[117] ClinicalTrials.gov U.S (2016). National library of medicine identifier NCT02660034 the safety, pharmacokinetics and antitumor activity of BGB-A317 in combination with BGB-290 in participants with advanced solid tumors. https://clinicaltrials.gov/ct2/show/NCT02660034

[118] ClinicalTrials.gov U.S (2018). National library of medicine identifier NCT03544125 olaparib and durvalumab in treating participants with metastatic triple negative breast cancer. https://clinicaltrials.gov/ct2/show/NCT03544125

[119] ClinicalTrials.gov U.S (2016). National library of medicine identifier NCT02849496 testing olaparib either alone or in combination with atezolizumab in BRCA mutant non-HER2-positive breast cancer. https://clinicaltrials.gov/ct2/show/NCT02849496

[120] ClinicalTrials.gov U.S (2022). National library of medicine identifier NCT05498155 study of neoadjuvant olaparib monotherapy and olaparib and durvalumab combination in HER2 negative brcam breast cancer (OlympiaN). https://clinicaltrials.gov/ct2/show/NCT05498155

[121] ClinicalTrials.gov U.S (2017). National library of medicine identifier NCT03025035 pembrolizumab in combination with olaparib in advanced BRCA-mutated or HDR-defect breast cancer. https://clinicaltrials.gov/ct2/show/NCT03025035

[122] ClinicalTrials.gov U.S (2017). National library of medicine identifier NCT03167619 phase II multicenter study of durvalumab and olaparib in platinum treated advanced triple negative breast cancer (DORA). https://clinicaltrials.gov/ct2/show/NCT03167619

[123] ClinicalTrials.gov U.S (2021). National library of medicine identifier NCT04837209 radiation, immunotherapy and PARP inhibitor in triple negative breast cancer (NADiR). https://clinicaltrials.gov/ct2/show/NCT04837209

[124] ClinicalTrials.gov U.S (2020). National library of medicine identifier NCT04673448 niraparib and TSR-042 for the treatment of BRCA-mutated unresectable or metastatic breast, pancreas, ovary, fallopian tube or Primary Peritoneal Cancer. https://clinicaltrials.gov/ct2/show/NCT04673448

[125] ClinicalTrials.gov U.S (2016). National library of medicine identifier NCT02657889 niraparib in combination with pembrolizumab in patients with triple-negative breast cancer or ovarian cancer (TOPACIO). https://clinicaltrials.gov/ct2/show/NCT02657889

[126] ClinicalTrials.gov U.S (2017). National library of medicine identifier NCT03101280 a combination study of rucaparib and atezolizumab in participants with advanced gynecologic cancers and triple-negative breast cancer. https://clinicaltrials.gov/ct2/show/NCT03101280

[127] ClinicalTrials.gov U.S (2019). National library of medicine identifier NCT04034927 testing the addition of an immunotherapy drug, tremelimumab, to the PARP inhibition drug, olaparib, for recurrent ovarian, fallopian tube or peritoneal cancer. https://clinicaltrials.gov/ct2/show/NCT04034927

[128] ClinicalTrials.gov U.S (2015). National library of medicine identifier NCT02571725 PARP-inhibition and CTLA-4 blockade in BRCA-deficient ovarian cancer full text view. https://clinicaltrials.gov/ct2/show/NCT02571725

[129] ClinicalTrials.gov U.S (2016). National library of medicine identifier NCT02953457 olaparib, durvalumab, and tremelimumab in treating patients with recurrent or refractory ovarian, fallopian tube or primary peritoneal cancer with BRCA1 or BRCA2 mutation. https://clinicaltrials.gov/ct2/show/NCT02953457

[130] ClinicalTrials.gov U.S (2019). National library of medicine identifier NCT03955471 study to evaluate the efficacy and safety of the combination of niraparib and dostarlimab (TSR-042) in participants with platinum resistant ovarian cancer (MOONSTONE). https://clinicaltrials.gov/ct2/show/NCT03955471

[131] ClinicalTrials.gov U.S (2018). National library of medicine identifier NCT03598270 platinum-based chemotherapy with atezolizumab and niraparib in patients with recurrent ovarian cancer (ANITA). https://clinicaltrials.gov/ct2/show/NCT03598270

[132] Domchek, S. M., Aghajanian, C., Shapira-Frommer, R., Schmutzler, R. K., Audeh, M. W. et al. (2016). Efficacy and safety of Olaparib monotherapy in germline BRCA1/2 mutation carriers with advanced ovarian cancer and three or more lines of prior therapy. Gynecologic Oncology*,* 140 *(*2*),* 199–203. 10.1016/j.ygyno.2015.12.020; 26723501 PMC4992984

[133] Martello, R., Mangerich, A., Sass, S., Dedon, P. C., Burkle, A. (2013). Quantification of cellular poly(ADP-ribosyl)ation by stable isotope dilution mass spectrometry reveals tissue- and drug-dependent stress response dynamics. ACS Chemical Biology*,* 8 *(*7*),* 1567–1575. 10.1021/cb400170b; 23631432 PMC3795969

[134] Telli, M. L., Timms, K. M., Reid, J., Hennessy, B., Mills, G. B. et al. (2016). Homologous recombination deficiency (HRD) score predicts response to platinum-containing neoadjuvant chemotherapy in patients with triple-negative breast cancer. Clinical Cancer Research*,* 22 *(*15*),* 3764–3773. 10.1158/1078-0432.CCR-15-2477; 26957554 PMC6773427

[135] Castroviejo-Bermejo, M., Cruz, C., Llop-Guevara, A., Gutiérrez-Enríquez, S., Ducy, M. et al. (2018). A RAD51 assay feasible in routine tumor samples calls PARP inhibitor response beyond BRCA mutation. EMBO Molecular Medicine*,* 10 *(*12*),* e9172. 10.15252/emmm.201809172; 30377213 PMC6284440

[136] Jacot, W., Thezenas, S., Senal, R., Viglianti, C., Laberenne, A. C. et al. (2013). BRCA1 promoter hypermethylation, 53BP1 protein expression and PARP-1 activity as biomarkers of DNA repair deficit in breast cancer. BMC Cancer*,* 13 *(*1*),* 523. 10.1186/1471-2407-13-523; 24191908 PMC4228368

[137] Ashworth, A. (2008). Drug resistance caused by reversion mutation. Cancer Research*,* 68 *(*24*),* 10021–10023. 10.1158/0008-5472.CAN-08-2287 19074863

[138] Christie, E. L., Fereday, S., Doig, K., Pattnaik, S., Dawson, S. J. et al. (2017). Reversion of BRCA1/2 germline mutations detected in circulating tumor DNA from patients with high-grade serous ovarian cancer. Journal of Clinical Oncology*,* 35 *(*12*),* 1274–1280. 10.1200/JCO.2016.70.4627; 28414925

[139] Rottenberg, S., Jaspers, J. E., Kersbergen, A., van der Burg, E., Nygren, A. O. et al. (2008). High sensitivity of BRCA1-deficient mammary tumors to the PARP inhibitor AZD2281 alone and in combination with platinum drugs. Proceedings of the National Academy of Sciences of the United States of America*,* 105 *(*44*),* 17079–17084. 10.1073/pnas.0806092105; 18971340 PMC2579381

[140] Choi, Y. E., Meghani, K., Brault, M. E., Leclerc, L., He, Y. J. et al. (2016). Platinum and PARP inhibitor resistance due to overexpression of microRNA-622 in BRCA1-mutant ovarian cancer. Cell Reports*,* 14 *(*3*),* 429–439. 10.1016/j.celrep.2015.12.046; 26774475 PMC4731274

[141] Du, Y., Yamaguchi, H., Wei, Y., Hsu, J. L., Wang, H. L. et al. (2016). Blocking c-Met-mediated PARP1 phosphorylation enhances anti-tumor effects of PARP inhibitors. Nature Medicine*,* 22 *(*2*),* 194–201. 10.1038/nm.4032; 26779812 PMC4754671

